# Sequentially Programmable and Cellularly Selective Assembly of Fluorescent Polymerized Vesicles for Monitoring Cell Apoptosis

**DOI:** 10.1002/advs.201700310

**Published:** 2017-08-10

**Authors:** Shu Peng, Yu‐Chen Pan, Yaling Wang, Zhe Xu, Chao Chen, Dan Ding, Yongjian Wang, Dong‐Sheng Guo

**Affiliations:** ^1^ College of Chemistry State Key Laboratory of Elemento‐Organic Chemistry Key Laboratory of Functional Polymer Materials Ministry of Education Nankai University Tianjin 300071 China; ^2^ Key Laboratory of Bioactive Materials Ministry of Education College of Life Sciences Nankai University Tianjin 300071 China; ^3^ Collaborative Innovation Center of Chemical Science and Engineering Nankai University Tianjin 300071 China

**Keywords:** bioimaging, cell apoptosis, enzymes, photopolymerization, programmable self‐assembly

## Abstract

The introduction of controlled self‐assembly into living organisms opens up desired biomedical applications in wide areas including bioimaging/assays, drug delivery, and tissue engineering. Besides the enzyme‐activated examples reported before, controlled self‐assembly under integrated stimuli, especially in the form of sequential input, is unprecedented and ultimately challenging. This study reports a programmable self‐assembling strategy in living cells under sequentially integrated control of both endogenous and exogenous stimuli. Fluorescent polymerized vesicles are constructed by using cholinesterase conversion followed by photopolymerization and thermochromism. Furthermore, as a proof‐of‐principle application, the cell apoptosis involved in the overexpression of cholinesterase in virtue of the generated fluorescence is monitored, showing potential in screening apoptosis‐inducing drugs. The approach exhibits multiple advantages for bioimaging in living cells, including specificity to cholinesterase, red emission, wash free, high signal‐to‐noise ratio.

## 1 Introduction

Construction of self‐assembled nanomaterials aiming to biomedical applications is attractive ever‐growing interests, including various biosensing/imaging probes, drug delivery systems, tissue engineering materials for the purpose of diagnosis and therapy.^1^, ^2^, ^3^, ^4^, ^5^ On demand of precise medicine with sensitive/selective diagnosis and targeting therapy, development of activatable theranostic nanoagents that can undergo an intrinsic evolution upon cell uptake is highly imperative.^6^, ^7^, ^8^, ^9^ Compared to the general way that self‐assembled nanoagents were beforehand produced in inanimate environments, the introduction of controlled self‐assembly into living things paves an alternative avenue to generate smart biomedical materials.^10^, ^11^, ^12^, ^13^ In these examples, molecular building blocks undergo self‐assembly following initial cellular uptake and subcellular activation.^14^, ^15^, ^16^ From the viewpoint of diagnostics, Rao's group imparted a biocompatible/bioorthogonal cyclization‐mediated in situ self‐assembly of small‐molecule probes for imaging protease activity both in vitro and in vivo.^17^, ^18^ Wang's group fabricated a novel photoacoustic contrast agent from an enzyme‐activated building block for specific and sensitive bacterial infection detection.^19^ From the viewpoint of therapeutics, enzyme‐instructed intracellular molecular self‐assembly exhibits superiorities of not only selectively killing cancer cells but also overcoming multidrug resistance,^20^, ^21^, ^22^ which was recently demonstrated by Xu's and Liang's groups concurrently.

**Scheme 1:**
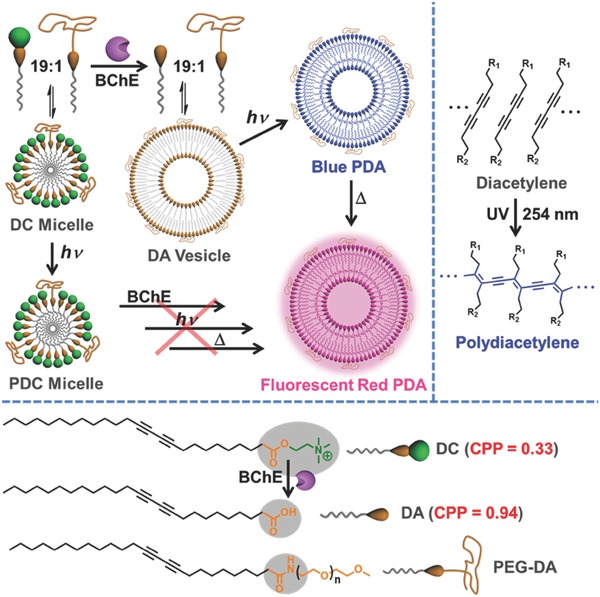
Schematic illustration of fabricating fluorescent polymerized vesicles (red PDA) from DC by a sequentially programmable control in the order of enzyme, light, and heating inputs, which cannot be otherwise achieved, even with the right combination of stimuli in different orders. BChE represents the abbreviation of butyrylcholinesterase. Methoxy poly(ethylene glycol)‐conjugated diacetylene (PEG‐DA) was doped on account of solubility, degree of hydration, stability, as well as thermochromism.

Enzymes, as an endogenous stimulus with desired specificity, have been commonly used to convert nonassembling precursors into self‐assembling blocks in living things,^14^, ^15^, ^16^, ^23^ which could alternatively achievable by some biocompatible exogenous stimuli too. However, self‐assembly under integrated control of both endogenous and exogenous stimuli, especially in the form of sequential input, is unprecedented and ultimately challenging. In this work, we wish to report a sequentially programmable self‐assembling strategy for fluorescent polymerized vesicles (**Scheme** **1**). To the best of our knowledge, this work represents the first example of programmable self‐assembly in living cells under sequentially integrated control of both endogenous and exogenous stimuli, i.e., enzymatic reaction and photopolymerization, respectively. Such programmable self‐assembly, relying on operations involving two or more stimuli arranged in a specific sequence,^24^, ^25^ can foreseeably improve selectivity and modularity of in situ and/or in vivo prepared smart biomaterials that can and will only respond to multiple stimuli in a predefined cascade.^26^, ^27^, ^28^ Moreover, fluorescence generation accompanied with the programmable self‐assembly endowed us capability of visualizing on site and in time information on biological structures and processes. Consequently, a proof‐of‐principle example of biomedical applications was demonstrated for monitoring the cell apoptosis process with cholinesterase as biomarker, which further allows apoptosis‐related drug screening. The fluorescent probe generated in situ by programmable self‐assembly exhibits multiple advantages, including specificity to cholinesterase, red emission over 600 nm, wash free, high signal‐to‐noise ratio for bioimaging in living cells.

## 2 Results and Discussion

To meet the requirement of enzyme‐responsivity in self‐assembly, the aggregation behaviors of enzymatic substrates and products have to be distinct from each other, e.g., enzymes convert nonassembling substrates into self‐assembling products or vice versa. The building precursor diacetylene‐appended choline (DC) forms the micellar assembly in the 20 × 10^−3^ m 4‐(2‐hydroxyethyl)piperazine‐1‐erhanesulfonic acid (HEPES) buffer (pH = 7.4, 150 × 10^−3^ m KCl) with a critical micelle concentration value of 14 µm (Figure S4, Supporting Information). The hydrolyzed product diacetylene acid (DA) has been well reported to form vesicular aggregation in aqueous solution.^29^, ^30^, ^31^ According to the calculation of critical packing parameter (CPP),^32^ DA with a CPP value of 0.94 prefers to form spherical vesicle while DC with a CPP value of 0.33 prefers to form spherical (or cylindrical) micelle.^33^ However, DA cannot be well hydrated in HEPES buffer even with harsh sonication at 80 °C for 30 min owing to its poor water solubility. Poly(ethylene glycol) (PEG) doping was then implemented to address this issue. Methoxy poly(ethylene glycol)‐conjugated diacetylene (PEG‐DA) was prepared according to a literature procedure.^34^, ^35^ Doping PEG‐DA to DA generates a DA vesicle coated with PEG on its surface. PEG incorporation can therefore improve the solubility of DA and make hydration of DA vesicle occur easily in mild conditions.^36^ A comprehensive screen of the molar ratio of PEG‐DA and DA shows that 5% PEG‐doping is optimal for operating the programmable self‐assembly in both inanimate environments and living cells on accounts of solubility, hydration, photopolymerization, and colorimetric response (see the Supporting Information).

**Figure 1:**
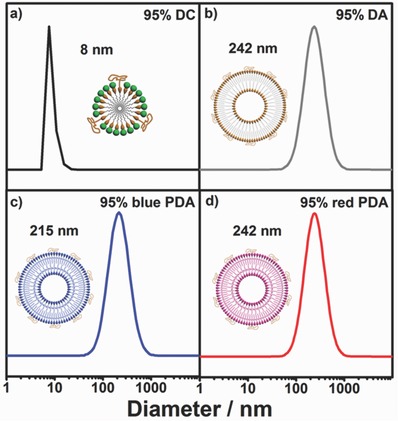
DLS data of a) 95% DC micelle, b) 95% DA vesicle, c) 95% blue PDA vesicle, and d) 95% red PDA vesicle in HEPES buffer (0.2 × 10^−3^ m in diacetylene unit).

Dynamic light scattering (DLS) and cryoelectron microscopy (cryo‐EM) and small‐angle X‐ray scattering (SAXS) measurements were employed to identify the different self‐assembling morphology and size between DC and DA. The DLS examinations revealed that 95% DC (5% PEG‐doping) forms small micellar aggregation, giving an averaged diameter of 8 nm, whereas 95% DA forms large vesicular aggregation with an averaged diameter of 242 nm (**Figure** **1**). UV irradiation of 95% DA for 5 min generates 95% blue polydiacetylene acid (PDA) with the diameter changing slightly to 215 nm. Heating transfers 95% blue PDA to the red one with a diameter of 242 nm. DLS and static light scattering measurements were combined to provide a profile of the assembling morphology as this is reflected by the ratio *R*_g_/*R*_H_.^37^ The ratio *R*_g_/*R*_H_ is calculated as 0.91 (Figure S10, Supporting Information), indicating the vesicular morphology. Transmission electron microscope (TEM) images show spherical morphology of both 95% DC and 95% polydiacetylene choline (PDC) and the sizes of 95% DC and 95% PDC are consistent with DLS results. (Figure S11, Supporting Information) Cryo‐EM images show the hollow spherical morphology of both 95% DA and 95% PDA, reinforcing the vesicular structure (Figure S12, Supporting Information). SAXS measurements give the same wall thickness of 4.7 nm for 95% DA and 95% PDA, which is in good accordance with two DA lengths (Figure S13, Supporting Information). All these results revealed that the enzyme product DA forms the large unilamellar vesicular aggregation whereas the enzyme substrate DC forms the small micellar aggregation. Neither the photopolymerization nor the thermochromism procedure affects the assembling size and morphology of DA vesicle appreciably.

**Figure 2:**
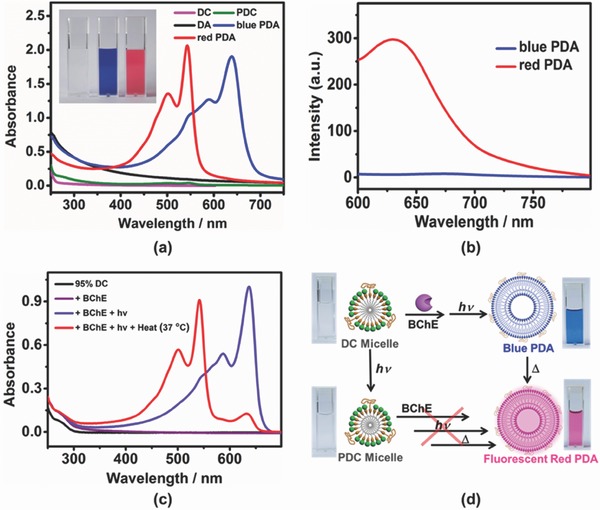
a) UV–vis spectra of DC, PDC, DA, blue PDA, and red PDA (0.2 × 10^−3^ m in diacetylene unit) in 10 × 10^−3^ m phosphate buffer saline (PBS,) (H = 8.0, considering the solubility of DA). PDC and blue PDA were produced by UV irradiation at 254 nm of DC for 5 h and DA for 5 min, respectively. Blue PDA was converted to red PDA when heated at 70 °C. The insets show the sequential color changes of the DA solution after photopolymerization and thermochromism. b) Fluorescence spectra of blue and red PDA in PBS buffer (0.2 × 10^−3^ m in diacetylene unit), λ_ex_ = 550 nm. c) UV–vis spectra of 95% DC in HEPES buffer (0.2 × 10^−3^ m in diacetylene unit) under the programmable control of the predefined cascade, where the stimuli are in the sequence of enzyme incubation (2 d), UV irradiation (5 min) and thermochromism. d) Schematic illustration showing the color changes of the 95% DC solution by multiple stimuli.

The distinguishable CPP values lead to not only the different assembly morphologies but also, more importantly, the distinct photopolymerization induced chromogenesis between DC and DA. After photopolymerization by UV irradiation, the color of DA vesicles turned deep blue (**Figure** **2**a), which is characteristic of a highly conjugated system, i.e., the formation of PDA. The blue polydiacetylene is susceptible to a wide range of external stimuli (temperature, pressure, light, etc.),^38^, ^39^, ^40^ leading to chromic changes.^29^, ^30^, ^41^, ^42^ The color of PDA changes from blue to carmine red when heated at 70 °C. Moreover, the colorimetric response of PDA from blue to red is accompanied by fluorescence generation (Figure 2b). Fluorescence, as one noninvasive optical technique, is preferable to observe the self‐assembly entities in living cells. In contrast to DA, photopolymerization cannot be obtained for DC with the same irradiation condition. Photopolymerization of DC was merely obtained when extending the irradiation time to several hours, forming PDC in pale yellow. The discriminative photopolymerization between DA and DC is ascribed to that the bilayer arrangement of DA vesicle is much more favorable for photopolymerization than the curvature arrangement of DC micelle.^43^, ^44^, ^45^, ^46^

For the process of programmable self‐assembly, the optimized 95% DC was subjected to the cascade stimuli, in the order of enzymatic hydrolysis, photopolymerization, thermochromism, monitored by UV–vis spectroscopy (Figure 2c). 5 U mL^−1^ butyrylcholinesterase (BChE) was employed according to the average activity of cholinesterase present in human tissues.^47^, ^48^ Incubation with BChE for 2 d converted DC to DA,^49^, ^50^, ^51^, ^52^ and then the DC micelles changed to the DA vesicles. Irradiation at 254 nm for 5 min led to the formation of the blue‐form PDA, and followed by a warming process at 37 °C, the desired fluorescent polymerized vesicles, red‐form PDA, were expectedly constructed, exhibiting red emission over 600 nm (Figure S14, Supporting Information).

The thermochromism kinetics at 37 °C was monitored in real time (Figure S15, Supporting Information), giving that the blue‐form PDA isomerized to the red form in a few minutes. The colorimetric response occurs quickly around body temperature (37 °C) to make the construction of fluorescent polymerized vesicles practically operational in living cells, which benefits from the PEG doping that could tune the colorimetric response temperature of PDA vesicles (Figure S8, Supporting Information, the PDA vesicle is more thermosensitive with PEG doping).^36^

The formation processes were further monitored by DLS (Figure S16, Supporting Information). As mentioned above, 95% DC shows very weak scattering intensity with the average diameter of 8 nm. After incubation with BChE, the scattering intensity increases dramatically, giving an average diameter of 96 nm, which definitely indicates the enzymatic conversion from the DC micelle to the DA vesicle. Further UV irradiation does not lead to appreciable size change (99 nm).^53^ Mass spectrometry (MS) and Zeta potential measurements also gave the indicative information about the enzymatic conversion. MS clearly shows the peak of choline, the other product of enzymatic hydrolysis, after incubation of DC with BChE (Figure S17a, Supporting Information). DC with the quaternary ammonium head group shows a positive Zeta potential of +36 mV, while DA with the carboxylic group shows a negative Zeta potential of −50 mV. Upon incubation with BChE, the Zeta potential of DC changes to −17 mV (Figure S18, Supporting Information).

To test the specificity of BChE‐triggered assembly, we investigated the programmable control of 95% DC by replacing BChE with other enzymes such as exonuclease I, trypsin, and α‐chymotrypsin (Figure S19a, Supporting Information). No photopolymerization induced chromogenesis was observed upon incubation with all control enzymes. To verify that the protein BChE itself is not a factor contributing to the chromogenesis, a control experiment was further carried out in which the same amount of denatured BChE (treated in boiling water for 1 h) was added to 95% DC, and no chromogenesis was observed either. Moreover, with the addition of tacrine, a BChE inhibitor, the BChE‐triggered chromogenesis was quenched to extent minimal level. Thus, the results clearly establish that it is the specific enzymatic activity of BChE, responsible for converting DC to DA, which serves as an indispensable prerequisite to build the fluorescent polymerized vesicles.

To test the necessity of programmable control, stimuli with an alternative sequence were imposed to 95% DC (Figure S19b, Supporting Information). When DC was imposed to UV irradiation first and then BChE incubation, followed by irradiation again, no appreciable chromogenesis was observed. The result demonstrates undoubtedly that implementing the multiple stimuli in the predefined cascade, that is, enzymatic hydrolysis followed by photopolymerization and thermochromism, is indispensable for fabricating the fluorescent polymerized vesicles while stimuli in any other sequence are invalid.

**Figure 3:**
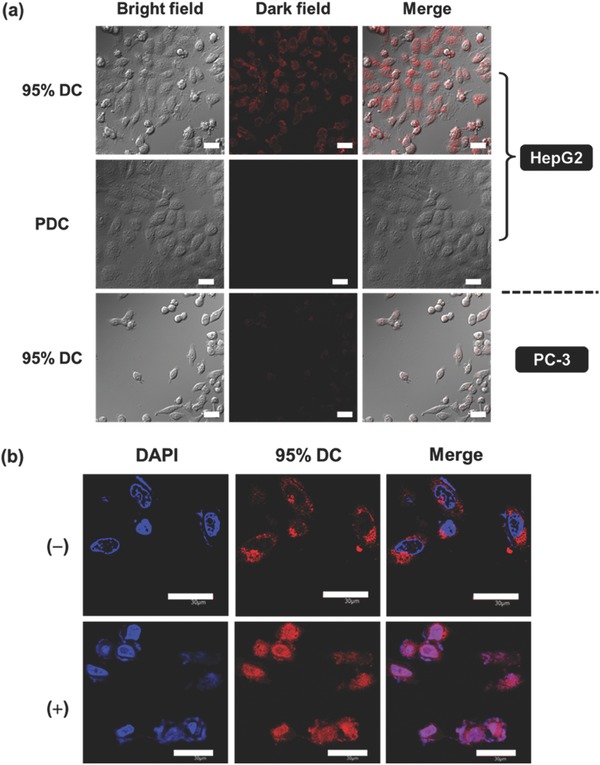
a) CLSM images of HepG2 cells incubated with 95% DC and 100% PDC, as well as PC‐3 cells incubated with 95% DC for 12 h at 37 °C (the concentration is 0.05 × 10^−3^ m in diacetylene unit), followed by UV irradiation. The scale bar is 40 µm. b) CLSM images of HepG2 cells incubated with 95% DC for 12 h at 37 °C without (−) or with (+) paclitaxel to trigger the cell apoptosis, followed by UV irradiation. The cell nuclei were stained with DAPI. The scale bar is 30 µm.

A cholinesterase‐rich cell line (HepG2) was then employed to evaluate the feasibility of the programmable self‐assembly strategy in living cells.^47^ It is prerequisite to examine the biocompatibility of the 95% DC micelles and the results show the low cell toxicity at experimental conditions (Figure S22, Supporting Information). The programmable fabrication of fluorescent polymerized vesicles in living cells was monitored by confocal laser scanning microscopy (CLSM). Fluorescent imaging shows the accumulation of bright red fluorescence inside cells (**Figure** **3**a). The fluorescence outside cells remains dim during the whole experiment, which provides a clear background and enables the visualization of the fabrication of fluorescent polymerized vesicles inside living cells.

It is clearly observed that intense red fluorescence was distributed in cytoplasm, whereas nearly no detectable signal was internalized by the cell nucleus, which implies that the fluorescent polymerized vesicles were generated in cytoplasm where cholinesterase is expressed. Figure 3b further validates the intracellular distribution of fluorescent polymerized vesicles, where the cell nuclei were costained with 4′,6‐diamidino‐2‐phenylindole (DAPI). The red fluorescence was mainly observed in cytoplasm around but not in nuclei. Note that no autofluorescence from the cell itself can be detected under the same experimental conditions (Figure S23, Supporting Information).

Notably, cholinesterase has been demonstrated as another kind of enzymatic biomarkers of cell apoptosis besides caspases.^54^, ^55^ Accompanying with the apoptosis process, cholinesterase is overexpressed and transfers from cytoplasm to cell nucleus.^56^ So far, assays for caspases have received considerable attention for apoptosis imaging;^18^, ^57^ however, developing cholinesterase assay principles for apoptosis imaging is still on demand. Only a few examples were reported^58^, ^59^ but suffered from puzzles of background fluorescence and washing difficulty. To our gratifying surprise, the present programmable self‐assembly is envisaged to be a smart strategy for imaging of live cell apoptosis with high fluorescence contrast. The precursor DC shows definitely no photopolymerization induced chromogenesis, which promises no interference of fluorescence background, and also, the fluorescent polymerized vesicles generated by cascade stimuli are light‐up probes with superiority of wash free. We therefore treated the cells with paclitaxel to trigger the cell apoptosis. Definitely, much brighter fluorescence was observed in the apoptotic cells than normal cells (Figure 3b). More importantly, the red fluorescence was observed in both cytoplasm and nucleus regions, indicating the transference of fluorescent polymerized vesicles from cytoplasm to cell nucleus. Both phenomena are indicative of the overexpression and transference of cholinesterase induced by apoptosis. As a result, the present system provides a new protocol for monitoring the cell apoptosis process involved in the overexpression of cholinesterase, which further allows in situ screening and quantification of apoptosis‐inducing agents. Comparing with the commercially available methods, it is a complementary approach to monitor cell apoptosis with cholinesterase as a biomarker, which may lead to a new understanding of mechanisms of apoptosis or drug action. Moreover, although sophisticated, the present strategy is still endorsed with multiple advantages, including specificity to cholinesterase, red emission, wash free, high signal‐to‐noise ratio for bioimaging in living cells.

As a blank control, the HepG2 cells were incubated with PDC, followed by the same operating procedure. No appreciable fluorescence was monitored by CLSM (Figure 3a), which is in accordance with the aforementioned result in the inanimate environments. That is, construction of fluorescent polymerized vesicles could be realized in both the inanimate environments and living cells, by only implementing the multiple stimuli in the predefined cascade, but not for any other undefined sequence of control.

Cholinesterase is also distributed in serum^47^ and 10% fetal bovine serum (FBS) is used in cell culturing process. It is therefore necessary to examine the influence of cholinesterase in cell medium on the programmable assembly process. 95% DC was incubated in cell medium at 37 °C for 12 h, and followed by the same operating procedure. No red fluorescence was observed (Figure S24, Supporting Information). This result reinforced that in cell experiments the fluorescent polymerized vesicles were fabricated in living cells but not in cell medium. Enzymatic conversion is the rate controlling process in constructing fluorescent polymerized vesicles, and such a programmable self‐assembly in living cells is cholinesterase dependent. We further examined the programmable self‐assembly strategy in PC‐3 cell line, which is relatively cholinesterase deficient.^60^ Comparing with HepG2 cells, very weak red fluorescence was observed in PC‐3 cells (Figure 3a). That is, the programmable construction of fluorescent polymerized vesicle is with cellular selectivity, depending on the activity of cholinesterase.

## 3 Conclusion

In summary, we reported a sequentially programmable self‐assembling strategy that fluorescent polymerized vesicles were constructed in not only inanimate milieu but also, more importantly, in living cells by implementing the programmable control in a strictly choreographed sequence of operations, that is, enzymatic conversion followed by photopolymerization and thermochromism. The synergistic contribution of endogenous enzymatic reaction and exogenous photopolymerization imparts the self‐assembling strategy not only with cellular selectivity but also promised high degree of spatial‐temporal control, which may find use in various biomedical applications, such as bioimaging/sensing, regulation and perturbation of cellular processes, tissue engineering and beyond. Moreover, the programmable self‐assembly was accompanied with fluorescence generation, which therefore demonstrated a novel assay principle for imaging of apoptosis and in situ evaluation of apoptosis‐inducing agents in living cells. The same programmable self‐assembling strategy may be amendable to other enzymatic targets, and opens up new avenues for generating smart biomaterials in vitro and in vivo.

## 4 Experimental Section

*Materials Preparation*: All chemicals used are reagent grade unless noted. 10,12‐Pentacosadiunoic acid, DA was purchased from Alfa Aesar. BChE was purchased from equine serum (246 U mg^−1^), trypsin (from bovine pancreas), and tacrine (9‐amino‐1,2,3,4‐tetrahydroacridine hydrochloride hydrate) were purchased from Sigma‐Aldrich. Exonuclease I was purchased from Takara. α‐Chymotrypsin was purchased from Aladdin. All of these were used without further purification. Methoxy PEG‐DA was synthesized and purified according to procedures reported previously.^34^, ^35^ The synthesis and characterization of DC were shown in the Supporting Information. FBS and Roswell Park Memorial Institute (RPMI) 1640 cell culture medium were purchased from Gibco. DAPI was purchased from Sigma.

*A General Procedure for the Fabrication of Assemblies*: Preparation of the DA vesicles: PEG‐DA was dissolved in buffer at a concentration of 1.0 × 10^−3^ m. A solution of 1.0 × 10^−3^ m DA in chloroform was dried for several hours under vacuum. Then a certain amount of PEG‐DA solution and buffer were added until final concentrations of 0.1 × 10^−3^ or 0.2 × 10^−3^ m in diacetylene unit. The samples were sonicated at 60−80 °C for 30 min, subsequently cooled to room temperature.

*Preparation of the DC Micelles*: A certain amount of PEG‐DA solution was added to the DC solution, and buffer was added until got final concentrations of 0.2 × 10^−3^ m in diacetylene unit. The sample was sonicated at 60 °C for 30 min and subsequently cooled to room temperature.

*A General Procedure for the Programmable Self‐Assembling Process in Inanimate Milieu*: 0.2 × 10^−3^ m 95% DC solution was prepared as described above. The solution was incubated with 5 U mL^−1^ BChE at 37 °C for 2 d to ensure enzymatic conversion DC to DA, and then was cooled at 4 °C overnight. The sample was sequentially irradiated with UV light (254 nm, 120 W) for 5 min at room temperature, leading to the formation of the blue‐form PDA, and followed by warming up to 37 °C for 15 min to generate the red‐form PDA.

*A General Procedure for the Programmable Self‐Assembling Process in Living Cells*: The HepG2 cells were cultured in Dulbecco's modified Eagle's medium (DMEM) each supplemented with 10% FBS in 5% CO_2_ at 37 °C. The PC‐3 cells were cultured with RPMI 1640, which was supplemented with 10% FBS, at 37 °C in a humidified atmosphere with 5% CO_2_. Both HepG2 and PC‐3 cells were incubated with 95% DC or 100% PDC (0.05 × 10^−3^ m in diacetylene unit) at 37 °C for 12 h and then were cooled at 4 °C for 2 h. The cells were sequentially irradiated with UV light (254 nm, holding‐lamp, 14.6 mJ cm^−2^) for 15 min at room temperature followed by warming up to 37 °C for a few minutes.

*A General Procedure for the DAPI‐Labeled Process*: The HepG2 cells were cultured in DMEM each supplemented with 10% FBS in 5% CO_2_ at 37 °C. HepG2 cells were incubated with 95% DC (0.05 × 10^−3^ m in diacetylene unit) at 37 °C for 12 h and then were cooled at 4 °C for 2 h. Alternatively, 0.05 × 10^−3^ m paclitaxel was added at 9 h to trigger the cell apoptosis within the 12 h DC incubation at 37 °C. The cells were sequentially irradiated with UV light (254 nm, holding‐lamp, 14.6 mJ cm^−2^) for 15 min at room temperature followed by warming up to 37 °C for a few minutes. HepG2 cells were washed three times with sterile 1 × PBS buffer and fixed using 1 mL 4% paraformaldehyde for 15 min at room temperature. After that, the HepG2 cells were washed with 1 × PBS buffer and treated with 1 mL of 0.1% Triton X‐100 for 10 min at room temperature. Then, the cells were also washed three times carefully and stained with DAPI for 10 min at room temperature. All cells were imaged by CLSM.

## Conflict of Interest

The authors declare no conflict of interest.

## Supporting information

Supplementary
