# Supplementary material for: Sequentially Programmable and Cellularly Selective Assembly of Fluorescent Polymerized Vesicles for Monitoring Cell Apoptosis
Source: Adv Sci (Weinh). 2017 Aug 10;4(11):1700310. doi: 10.1002/advs.201700310 (PMC5700639; doi:10.1002/advs.201700310)
Supplement: Supplementary file 1 — Supplementary [file ADVS-4-na-s001.pdf]

Copyright WILEY-VCH Verlag GmbH & Co. KGaA, 69469 Weinheim, Germany, 2017.

Supporting Information

for *Adv. Sci.,* DOI: 10.1002/advs.201700310

Sequentially Programmable and Cellularly Selective Assembly of Fluorescent Polymerized Vesicles for Monitoring Cell Apoptosis

Shu Peng, Yu-Chen Pan, Yaling Wang, Zhe Xu, Chao Chen, Dan Ding, Yongjian Wang,* and Dong-Sheng Guo**

Copyright WILEY-VCH Verlag GmbH & Co. KGaA, 69469 Weinheim, Germany, 2016.

Supporting Information

Sequentially Programmable and Cellularly Selective Assembly of Fluorescent Polymerized Vesicles for Monitoring Cell Apoptosis

Shu Peng, Yu-Chen Pan, Yaling Wang, Zhe Xu, Chao Chen, Dan Ding, Yongjian Wang,* and Dong-Sheng Guo**

Table of contents

1. Experimental Section S3

1.1 Synthesis of diacetylene-appended choline (DC) (Scheme S1 and Figures S1–S3) S3

1.2 Measurements S5

2. The self-assembly of enzymatic substrate DC and product DA (Figures S4 and S5) S7

3. Screening the PEG-doping ratio to DA S9

3.1 The photopolymerization performance of DA with different PEG-doping ratios (Figures S6 and S7) S9

3.2 The temperature-dependent colorimetric response of PDA with different PEG-doping ratios (Figures S8 and S9) S10

4. The self-assembly morphologies and sizes of 95% DA and PDA (Figures S10–S13) S14

5. Programmable construction of fluorescent polymerized vesicles from DC in inanimate environments (Figures S14–S19) S16

6. The influence of enzymatic reaction time on programmable assembly processes (Figures S20 and S21) S19

7. Programmable construction of fluorescent polymerized vesicles from DC in living cells (Figure S22–S24) S21

8. References S22

1. Experimental Section

1.1 Synthesis of diacetylene-appended choline (DC)

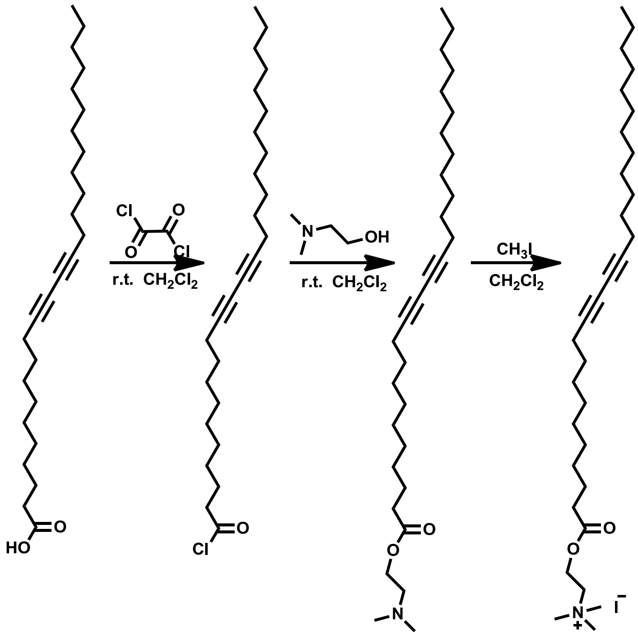

Scheme S1. Synthetic route of diacetylene-appended choline (**DC**).

Oxalyl chloride (0.14 mL, 1.6 mmol) in CH_2Cl_2 (5 mL) was added to a solution of **DA** (500 mg, 1.33 mmol) in CH_2Cl_2 (10 mL) at 0 °C. After stirring for 2 h at room temperature, the solvent and surplus oxalyl chloride was removed in vacuo and the residue was dissolved in CH_2Cl_2 (10 mL). Then, the solution was added N,N-dimethylethanolamine (0.13 mL, 1.33 mmol) and triethylamine (0.28 mL, 2 mmol) at 0 °C. The mixture solution was stirred over night at room temperature. The mixture was washed with saturated Na_2CO_3, water and brine. After drying with Na_2SO_4, the solvent was removed in vacuo and the residue was purified by column chromatography over silica gel with CH_2Cl_2/MeOH = 1/0 to 1/1 as the eluents to give diacetylene-appended dimethylethanolamine (460 mg, 77%). To the CH_2Cl_2 solution of diacetylene-appended dimethylethanolamine (460 mg, 1.03 mmol) CH_3I (0.096 mL, 1.5 mmol) was added, and the mixture solution was stirred over night at room temperature. The solvent was removed in vacuo and the residue was purified by column chromatography over silica gel with CH_2Cl_2/MeOH = 200/1 to 20/1 as the eluents to give **DC** (504 mg, 83%). ^{1}H NMR (400 MHz, $CDCl_3$, *δ*): 4.62–4.56 (m, 2H), 4.17–4.12 (m, 2H), 3.58 (s, 9H), 2.38 (t, J = 7.6 Hz, 2H), 2.24 (t, J = 6.9 Hz, 4H), 1.66–1.58 (m, 2H), 1.56–1.47 (m, 4H), 1.38–1.22 (m, 26H), 0.88 (t, J = 6.8 Hz, 3H); ^{13}C NMR (100 MHz, $CDCl_3$, *δ*) 172.72, 77.68, 77.47, 65.26, 57.64, 54.81, 34.09, 31.93, 29.63, 29.48, 29.35, 29.07, 28.89, 28.76, 28.29, 24.61, 22.70, 19.20, 14.14; ESI-FTMS (*m/z*): $[M–I]^+$ calcd. for $C_{30}H_{54}NO_2^+$ 460.4149, found 460.4154.

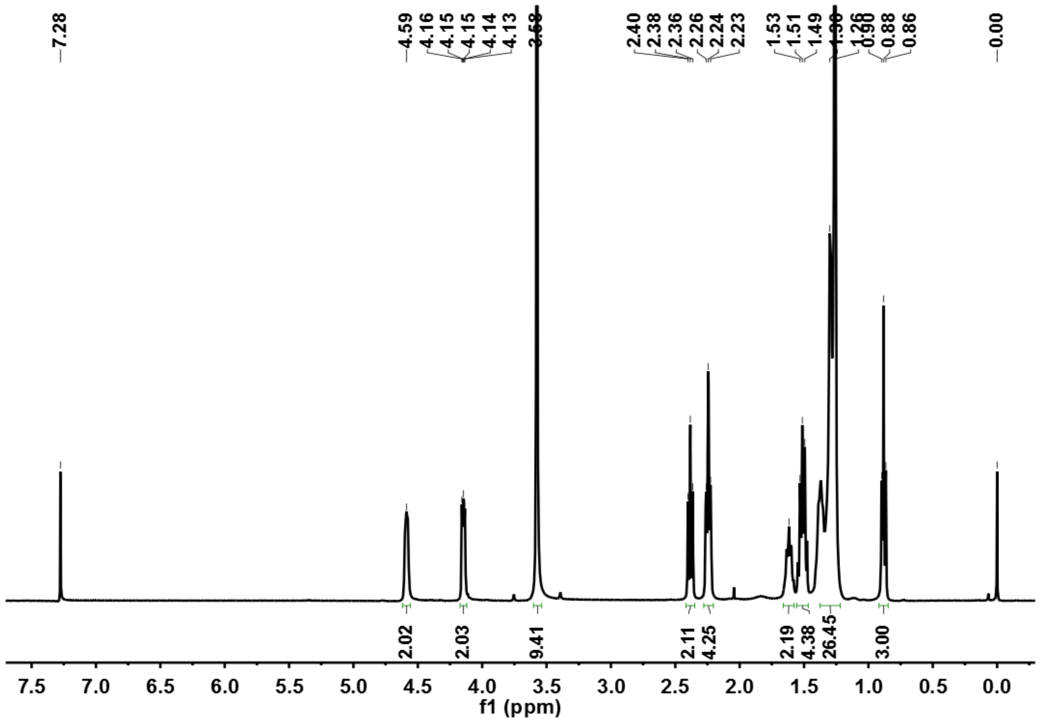

Figure S1. ^{1}H NMR spectrum of **DC** in $CDCl_3$ at 25 °C.

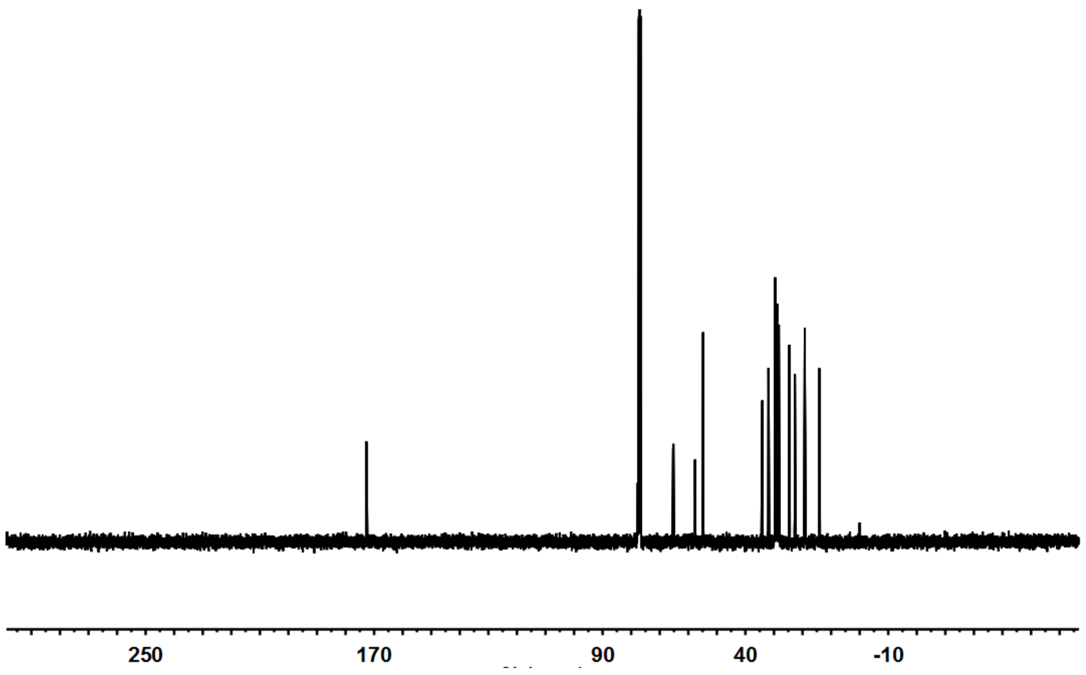

Figure S2. ^{13}C NMR spectrum of **DC** in $CDCl_3$ at 25 °C.

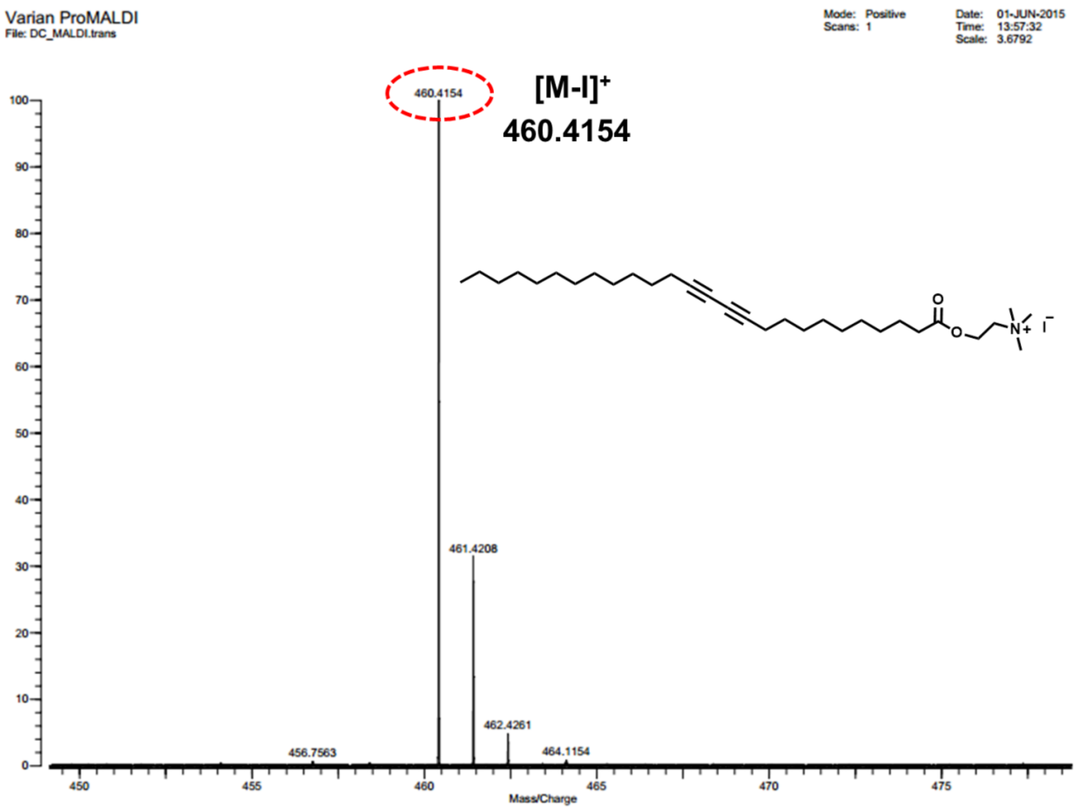

Figure S3. Electrospray ionization fourier transform ion cyclotron resonance-MS of **DC**.

1.2 Measurements

pH measurements

Preparation of the HEPES buffer (20 mM, 150 mM KCl, pH = 7.4): Dissolve 4.766 g of HEPES free acid in approx. 900 ml of double-distilled water. Titrate to pH 7.39 at the room temperature of 25 °C with KOH. Make up volume to 1000 ml with double-distilled water. Preparation of the PBS buffer (10 mM, pH = 8.0): Dissolve 1.56 g of $NaH_2PO_4{\cdot}2H_2O$ in approx. 900 ml of double-distilled water. Titrate to pH 8.0 at the room temperature of 25 °C with NaOH. Make up volume to 1000 ml with double-distilled water.

The pH values of PBS and HEPES buffers were measured on a Orion 3 Star Benchtop pH meter from Thermo Scientific.

UV-Vis spectroscopy

The optical absorbance of the solutions were measured in a quartz cell (light path 10 mm) on a Shimadzu UV-3600 spectrophotometer equipped with a PTC-348WI temperature controller.

Fluorescence spectroscopy

Fluorescence spectra were recorded in a conventional quartz cell (light path 10 mm) on a Varian Cary Eclipse equipped with a Varian Cary single-cell peltier accessory to control

temperature.

Dynamic light scattering (DLS) measurements

Angle-dependent DLS experiments were examined on a laser light scattering spectrometer (BI-200SM) equipped with a digital correlator (TurboCorr) at 636 nm. The others were measured by NanoBrook 173 plus at scattering angle of 90°.

Cryo-electron microscopy (Cryo-EM) experiments

Aliquots of 3.5 μL sample were applied to glow-discharged Quantifoil grids, blotted for 6s in a room temperature and 100% humidity chamber, and plunged into liquid ethane cooled by liquid nitrogen in the automated EFI Vitrobot device. We performed structural analysis by cryo-electron microscopy, on the FEI Talos F200C with constant-power C-Twin objective lens which was equipped with a Gatan Model 626 cryo-transfer specimen holder, operated at 200 kV. Images were recorded on the FEI 16Megapixel Ceta CMOS camera. The electron dose for each micrograph was approximately 30 e/Å^2.

Small-angle X-ray scattering (SAXS) measurements

SAXS experiments were performed with the high-flux small-angle X-ray scattering instrument (SAXSess, Anton Paar) equipped with a Kratky block collimation system and a Philips PW3830 sealed-tube X-ray generator (Cu Kα).

Zeta potential measurements

Zeta potential measurements were measured by NanoBrook 173 plus.

Nuclear magnetic resonance (NMR) spectroscopy

^{1}H NMR and ^{13}C NMR spectra were recorded on a Bruker AV400 spectrometer.

Mass spectroscopy (MS)

High resolution-MS measurements were recorded on a VG ZAB-HS mass spectrum. Electrospray ionization fourier transform ion cyclotron resonance-MS measurement was recorded on a Varian 7.0T FTICR-MS.

Confocal laser scanning microscopy (CLSM)

HepG2 and PC-3 cells incubated with target molecules in petri dish were observed by a confocal laser scanning microscope (Olympus FV1000). The programmable construction of fluorescent polymerized vesicles in living cells was monitored by confocal laser scanning microscopy. The experimental details are reported as **1.5** and **1.6**.

2. The self-assembly of enzymatic substrate DC and product DA

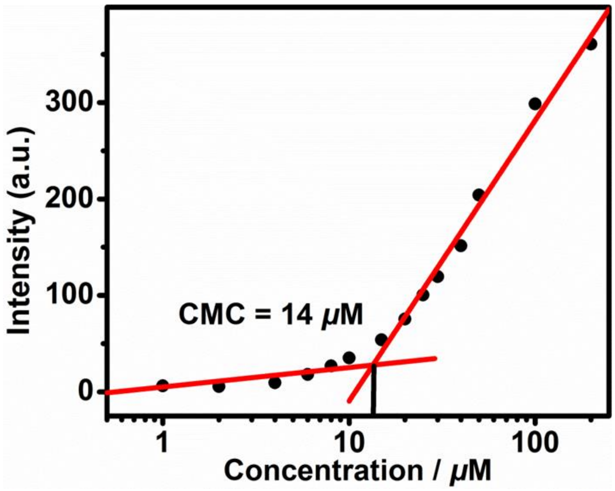

Figure S4. The critical micelle concentration of **DC** in HEPES buffer by nile red probe.

The hydrophobic parts of **DC** and **DA** are almost same, and therefore the key parameter controlling self-assembly morphology is the head group. The carboxy group in **DA** is smaller than the quaternary ammonium group in **DC**. Caborxy head groups afford weaker charge repulsion than quaternary ammonium head groups because it is a weak acid and only partly ionized. Hydrogen bonds are reasonably formed between carboxy groups and water molecules on the surface of **DA** assembly. All these factors facilitate a decrease in the lift-off area of **DA** (22 Å^2 of **DA** versus 65 Å^2 of **DC**). CPP is calculated by CPP = $V/(L\times A)$, where V is the alkyl chain volume, L is the length, A is the lift-off area per surfactant head group. In a general rule, CPP < 0.33, spherical micelle; CPP = 0.33–0.50, cylindrical micelle; CPP = 0.50–1.0, spherical vesicle; CPP = 1.0, planner bilayer; CPP > 1.0, reversed micelle.

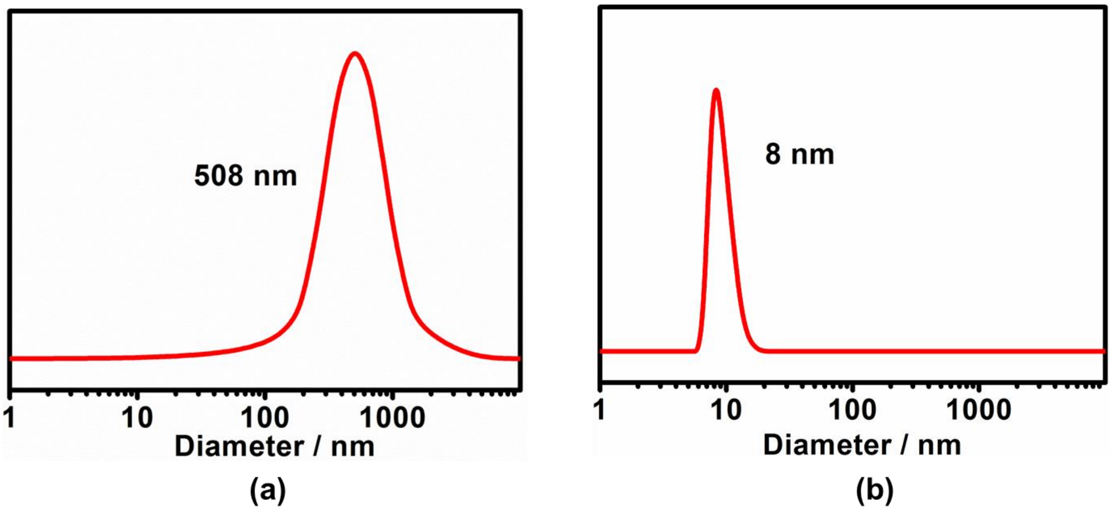

Figure S5. DLS data of (a) the **DA** vesicle (0.2 mM in diacetylene unit) and (b) the **DA-PEG** micelle (0.1 mM in diacetylene unit) in HEPES buffer.

DA cannot be well hydrated in HEPES buffer even with harsh sonication at 80 °C for 30 min. DLS measurement shows giant particles with an averaged diameter of 508 nm (Figure S5a), and the suspension precipitated in a few hours, probably because of the poor solubility of **DA** in neutral media. Poly(ethylene glycol) (**PEG**) doping was then implemented to address this issue. Doping **PEG-DA** to **DA** generates a **DA** vesicle coated with **PEG** on its surface. **PEG** incorporation can therefore improve the solubility of **DA**, and make hydration of **DA** vesicle occur easily in mild conditions.[1]

3. Screening the PEG-doping ratio to DA

3.1 The photopolymerization performance of DA with different PEG-doping ratios

It is a prerequisite to ascertain a molar ratio of **PEG-DA** and **DA** that ensures not only the solubility and stability but also the photopolymerization. Excessive **PEG** would result in assembling morphology transition as well as reduced photopolymerization because **PEG-DA** forms micellar assembly in aqueous media without any chromogenic property in response to UV irradiation. We examined the assembling and photopolymerization properties of **PEG-DA** in HEPES buffer. **PEG-DA**, similar to **DC**, forms micelles with an averaged diameter of 8 nm (Figure S5b), and no appreciable chromogenesis was observed upon UV irradiation (Figure S6). We therefore screened the doping stoichiometry between **PEG-DA** and **DA** by monitoring the chromogenic performance after UV irradiation (Figure S6a). When doping **PEG-DA** into **DA**, the hydration could be successfully operated under mild conditions, where the suspension became translucent soon after sonication. And also, the **PEG**-coated **DA** vesicles are stable enough to achieve a shelf life of several weeks at either 4 °C or room temperature. However, we noticed that the photopolymerization induced chromogenesis performance of **DA** decreases gradually along with increasing the **PEG-DA** ratio (Figure S6b). It is reasonably acceptable because **PEG-DA** itself tends to form micelles without showing any chromogenesis by UV irradiation. Consequently, coating **DA** vesicles with as little **PEG** as possible should be adopted to maintain high-performance chromogenesis. We further reduced the **PEG-DA** ratios to 1%, 2.5%, and 5% in all three cases of which satisfactory hydration and photopolymerization were successfully achieved (Figure S7). However, the hydration of 99% and 97.5% **DA** vesicles (corresponding to 1% and 2.5% **PEG** doping) occurs merely under a relatively harsh condition (sonication at 80 °C for 30 min), which is not biocompatible, and therefore, 1% and 2.5% **PEG** incorporations were excluded for fabricating the designed assembling entities in living cells.

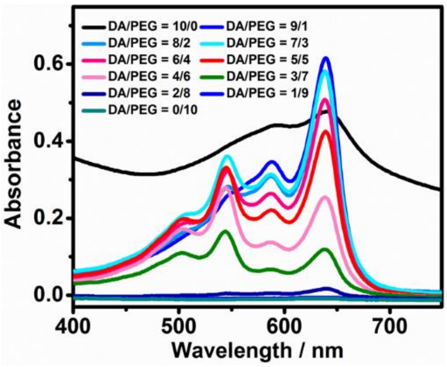

(a)

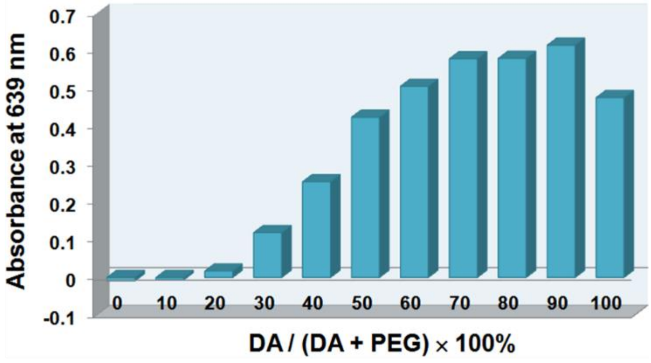

(b)

Figure S6. UV-Vis spectra of polydiacetylene assemblies of mixing **DA** and **PEG-DA** with different molar ratios in HEPES buffer (0.1 mM in diacetylene unit). The photopolymerization was achieved by UV irradiation at 254 nm for 10 min.

The absorption spectrum of ***DA*** *without* ***PEG*** *is obviously different from the others. The nonspecific optical density is indicative of light scattering by large-sized suspended particles, which further validated the inferior hydration of pure* ***DA*** *in HEPES buffer.*

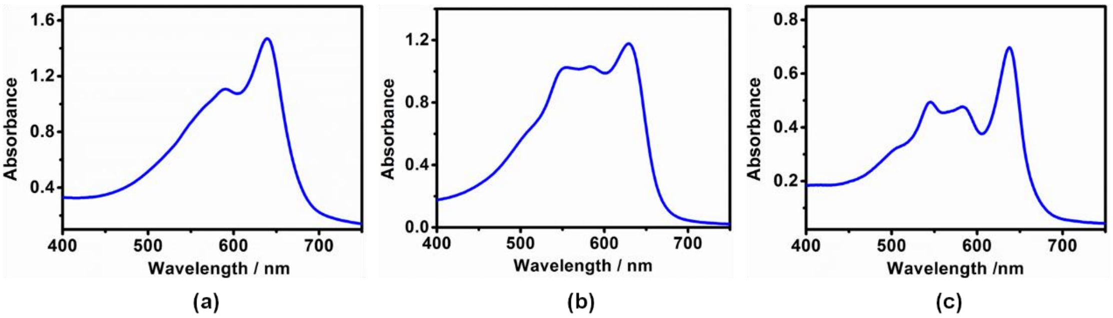

Figure S7. UV-Vis spectra of (a) 99% **PDA**, (b) 97.5% **PDA** and (c) 95% **PDA** in HEPES buffer at 25 °C (0.2 mM in diacetylene unit, 5–7 min irradiation).

3.2 The temperature-dependent colorimetric response of PDA with different PEG-doping ratios

Another important factor that should be of concern is the colorimetric response (CR) temperature of the polymerized vesicles. CR is defined as the percentage from blue to red. In this context, B value was defined as (A_{blue} / [A_{blue} + A_{red}]) × 100%, where A is the peak intensity corresponding to the blue or red form. CR was then calculated according to the initial and final B values, CR = ($B_{initial}$ − B_{final}) / $B_{initial}$ × 100%.[2] As mentioned in the main text, the blue **PDA** changes completely its color to red when heated at 70 °C. It is ideal that the color change occurs around body temperature (37 °C) to make the construction of fluorescent polymerized vesicles practically operational in living cells. A fascinating phenomenon is that **PEG** doping could tune the CR temperature of the **PDA** vesicles.[1] The CR and fluorogenic temperatures of **PDA** vesicles with different **PEG**-doping ratios (100% in PBS buffer, 99%, 97.5% 95%, 90% in HEPES buffer) were comparatively examined (Figures S8 and S9). Obviously, both the CR and fluorogenic sensitivity in response to heat increased with enhancing the **PEG**-doping ratios. 100% and 99% **PDA** vesicles show nearly complete color change at about 65 °C, as well as the fluorogenic property. The CR temperatures of 95% and 90% **PDA** vesicles appear at about 40 °C. As a result, **PEG** doping exhibits immense

effect of improving thermosensitivity of **PDA** vesicles, attributable to the assumption that the isomerization of **PDA** framework is more fragile to heat. Excessive **PEG** doping seems to be unnecessary, which would also jeopardize the chromogenic performance. *Consequently, 5%* ***PEG*** *doping sufficiently meet the requirement of building fluorescent polymerized vesicles in biological milieu from the viewpoints of mild hydration and optical CR temperature.* To summarize, coating **PDA** vesicles with **PEG** has been demonstrated to exhibit the following rewards: (1) enhancing water-solubility; (2) hydration in mild conditions; (3) improving mechanical stability from coagulation and settling; (4) tuning CR and fluorogenic temperature to around body temperature; (5) protection from the nonspecific absorption of disruptive proteins, which is crucial for further biological applications.[3]

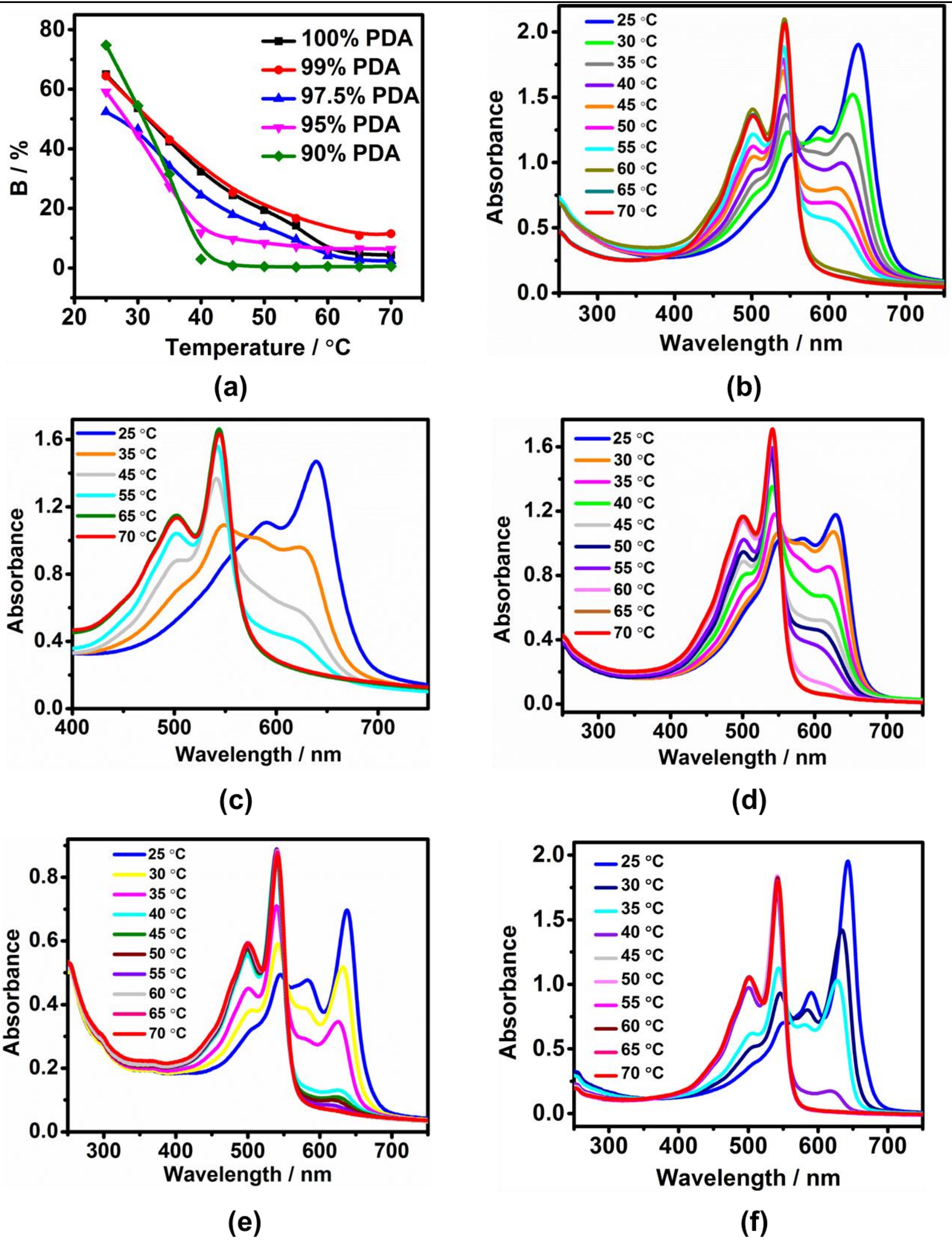

Figure S8. (a) Dependence of the corresponding B values of 100% PDA in PBS buffer, 99%, 97.5%, 95%, 90% PDA in HEPES buffer on temperature. Temperature-dependent UV-Vis spectra of (b) 100% **PDA** in PBS buffer, (c) 99% **PDA** in HEPES buffer, (d) 97.5% **PDA** in HEPES buffer, (e) 95% **PDA** in HEPES buffer and (f) 90% **PDA** in HEPES buffer (0.2 mM in diacetylene unit, 5–7 min irradiation).

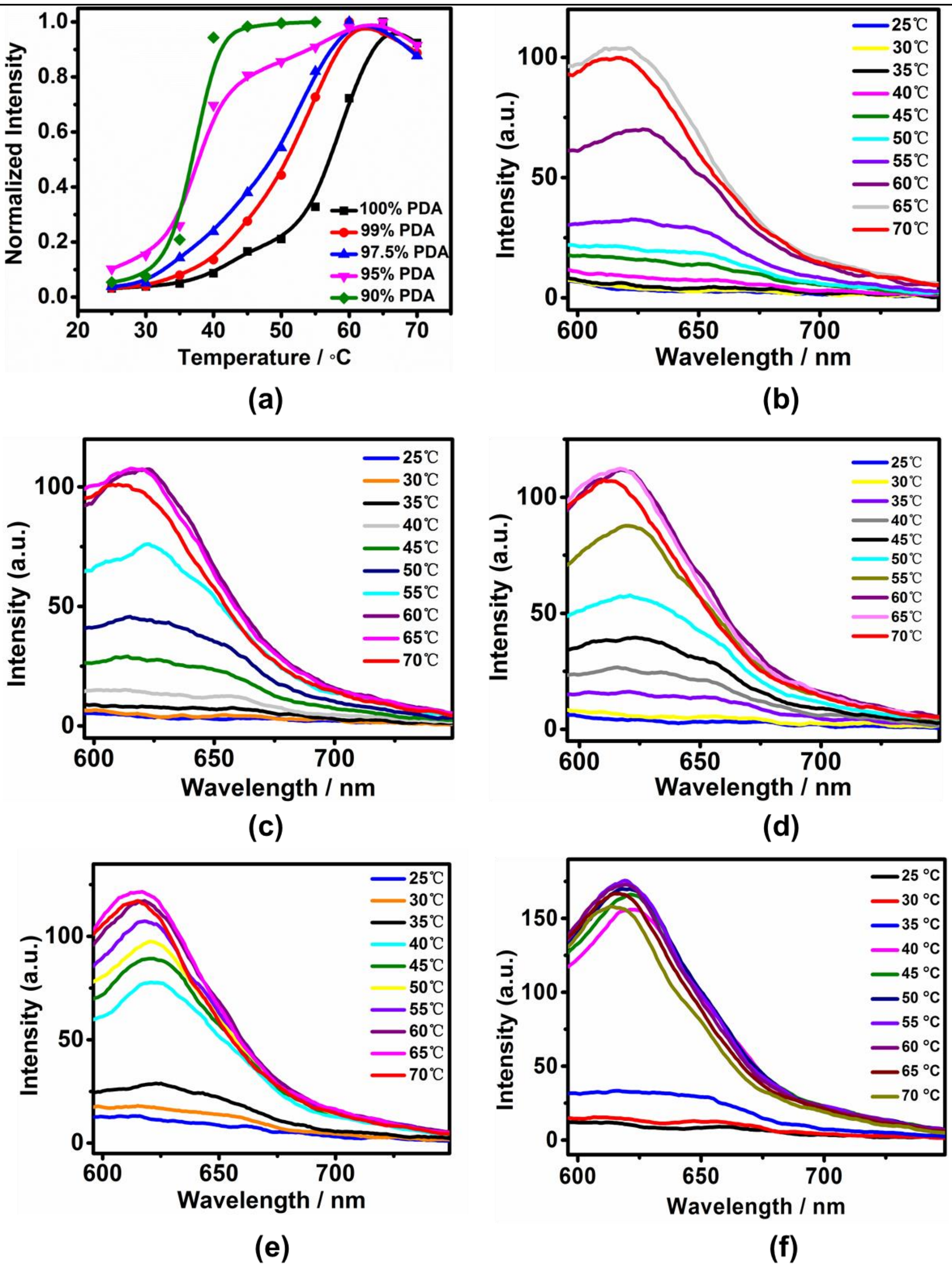

Figure S9. (a) Dependence of fluorescence intensities of 100% **PDA** in PBS buffer, 99%, 97.5%, 95%, 90% **PDA** in HEPES buffer on temperature. Temperature-dependent fluorescence spectra of (b) 100% **PDA** in PBS buffer, (c) 99% **PDA** in HEPES buffer, (d) 97.5% **PDA** in HEPES buffer, (e) 95% **PDA** in HEPES buffer and (f) 90% **PDA** in HEPES buffer (0.2 mM in diacetylene unit, 5 min irradiation).

4. The self-assembly morphologies and sizes of 95% DA and PDA

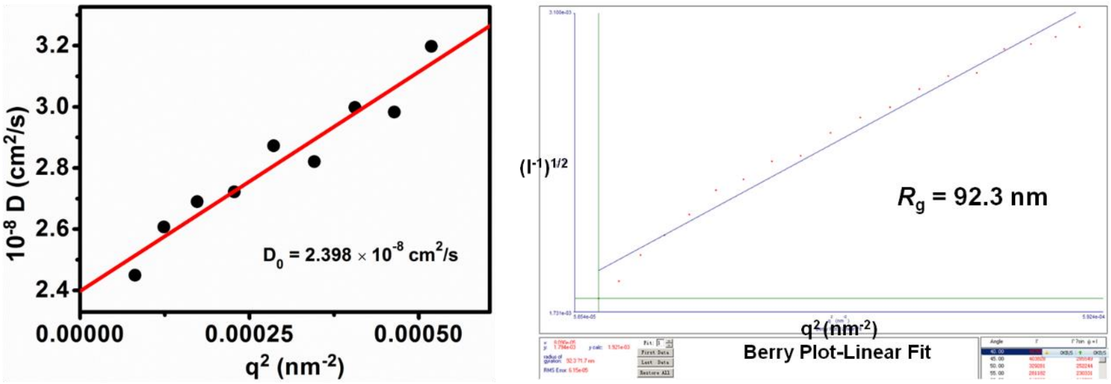

(a) (b)

Figure S10. (a) Dependence of the q^2 on quadratic diffusion coefficient of the 95% **DA** assembly at different angles ($40 \le \theta \le 130°$) in HEPES buffer (0.2 mM in diacetylene unit). (b) SLS data of the 95% **DA** assembly in HEPES buffer (0.2 mM in diacetylene unit).

Particles in solution move under Brownian motion and their diffusion coefficient D can be related to their (hydrodynamic) size by the Stokes–Einstein equation ($R_H = k_BT/6\pi\eta D$). In Figure S10a, the diffusion coefficient D was obtained. According to Stokes–Einstein equation, angle-dependent DLS experiments over a scattering angular range of 40−130° show an averaged hydrodynamic radius (R_H) of 101.7 nm of 95% **DA** (Figure S10a). SLS under the same conditions ($40 \le \theta \le 130°$) gives a gyration radius (R_g) of 92.3 nm (Figure S10b). The ratio R_g/R_H is calculated as 0.91.

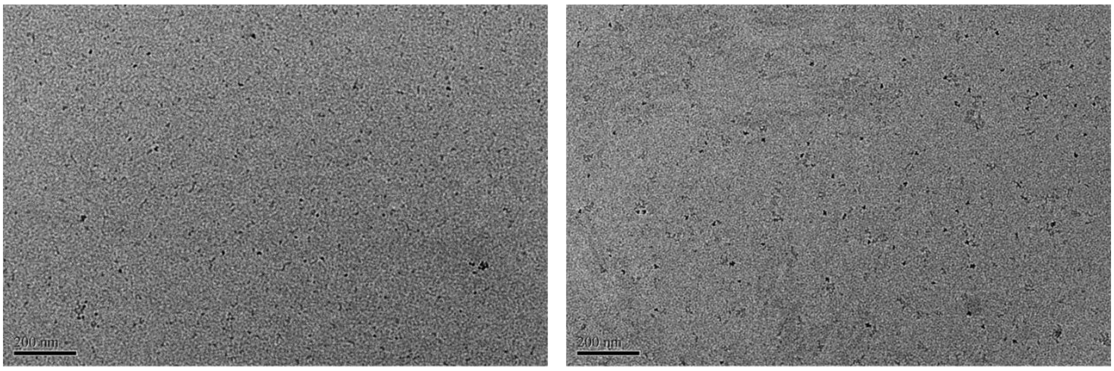

(a) (b)

Figure S11. TEM images of (a) 95% **DC** (b) 95% **PDC**.

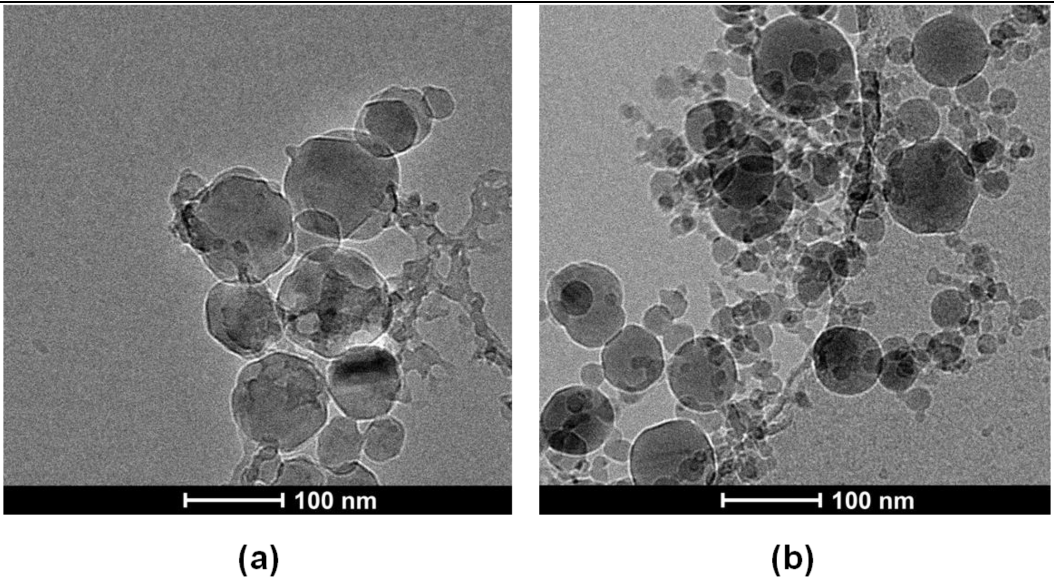

Figure S12. Cryo-EM images of (a) 95% **DA** (b) 95% **PDA** vesicles.

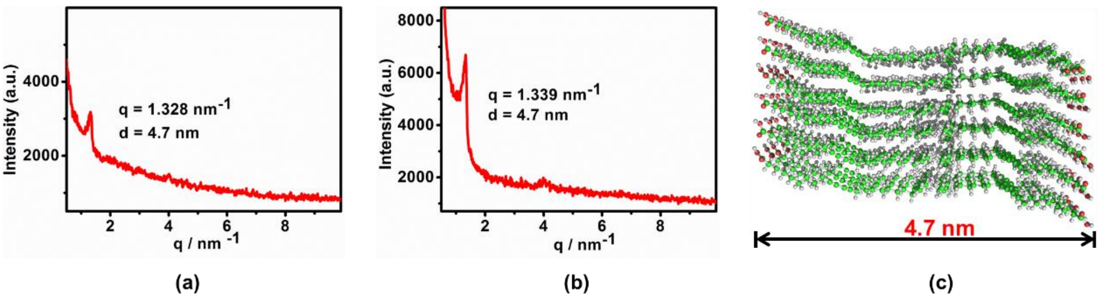

Figure S13. SAXS experiments of (a) 95% **DA** and (b) 95% **PDA**. (c) Packing structure showing the bilayer aggregation of **DA** vesicle.

5. Programmable construction of fluorescent polymerized vesicles from DC in inanimate environments

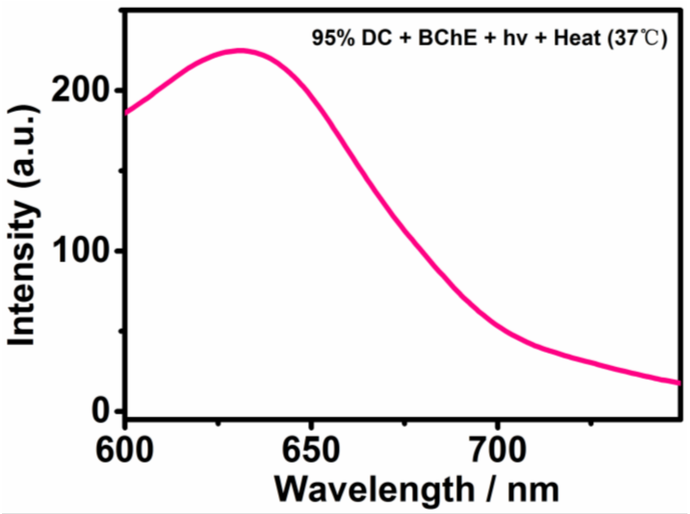

Figure S14. (c) Fluorescence spectrum of 95% **DC** in HEPES buffer (0.2 mM in diacetylene unit) with the stimuli in the sequence of enzyme incubation (2 days), UV irradiation (5 min) and thermochromism (37 °C), λ_{ex} = 550 nm.

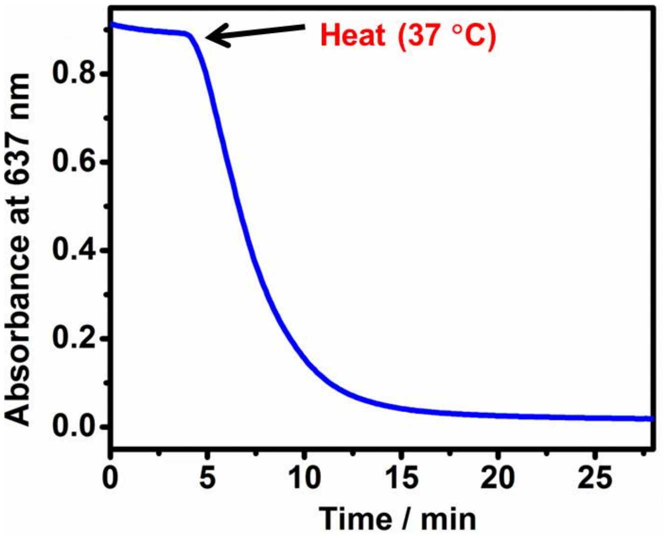

Figure S15. The thermochromism kinetics of 95% **PDA** in HEPES buffer at 37 °C monitored in real time (0.2 mM in diacetylene unit).

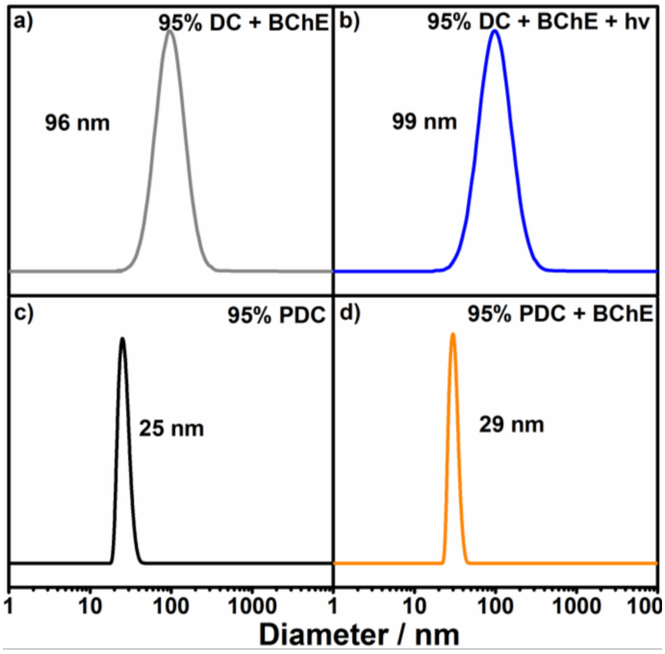

Figure S16. DLS data of (a) 95% **DC** upon incubation of 2 days with BChE and (b) UV irradiation of 5 min, (c) 95% **PDC**, (d) 95% **PDC** after incubation with BChE (0.2 mM in diacetylene unit).

In a reference sequence, the photopolymerization of **DC** by UV irradiation formed **PDC**, and then **PDC** was incubated with BChE, which is expected to produce oligomeric **PDA**. MS shows obvious choline signal after incubation of **PDC** with BChE (Figure S17b). However, DLS results showed that both 95% **PDC** before and after incubation with BChE formed merely the small micellar aggregation (Figure S16). We supposed that the oligomeric **PDA**, unlike **DA**, was incapable of forming well-defined bilayer arrangement necessary for photopolymerization. Consequently, implementing the multiple stimuli in the predefined cascade is necessary for fabricating the fluorescent polymerized vesicles while stimuli in any other sequence are invalid.

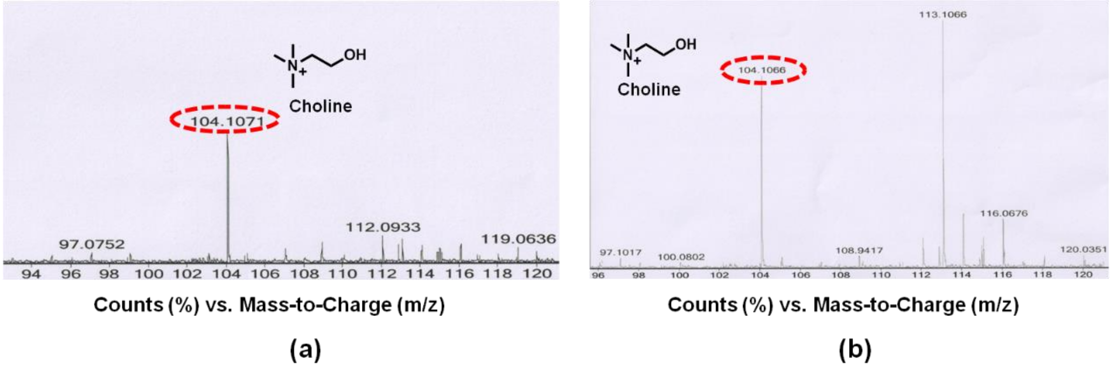

Figure S17. High resolution-MS of (a) **DC** and (b) **PDC** after incubation with BChE at 37 °C in PBS buffer for 2 days (0.2 mM in diacetylene unit).

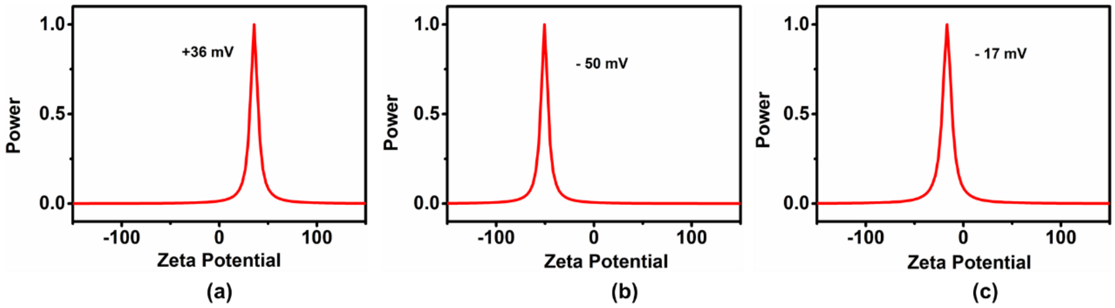

Figure S18. Zeta potential data of (a) **DC** (0.2 mM in diacetylene unit), (b) **DA** (0.1 mM in diacetylene unit) and (c) **DC** after incubation with BChE in PBS buffer (0.1 mM in diacetylene unit).

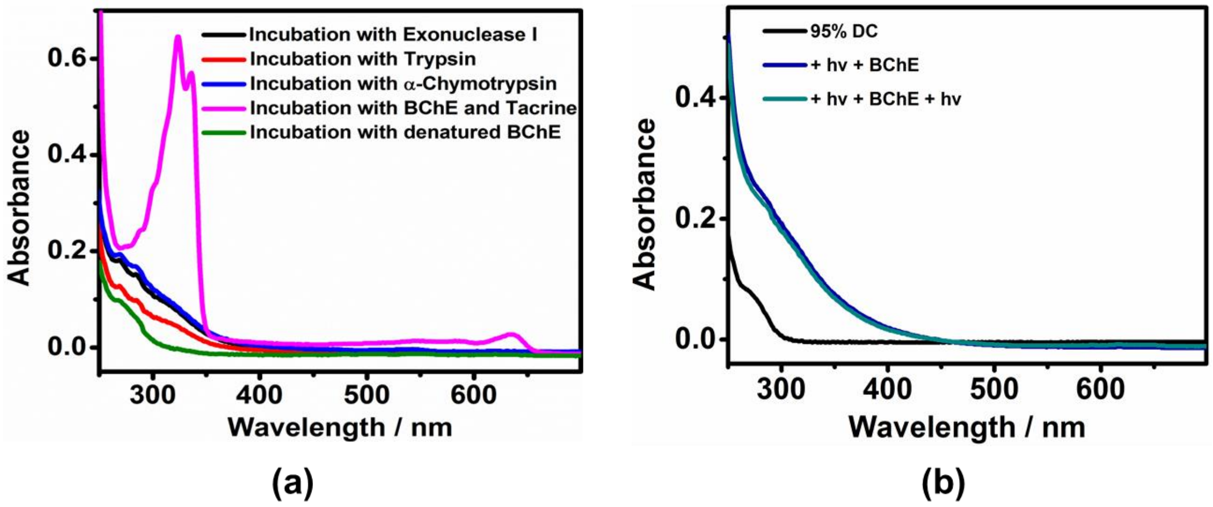

Figure S19. (a) UV-Vis spectra of 95% **DC** in HEPES buffer (0.2 mM in diacetylene unit) upon incubation (2 days) with exonuclease I, trypsin, α-chymotrypsin, BChE+tacrine and denatured BChE, followed by UV irradiation (5 min). (b) UV-Vis spectra of 95% **DC** in HEPES buffer (0.2 mM in diacetylene unit) under the control sequence. In a control sequence, the stimuli are in the sequence of UV irradiation (3 h), enzyme incubation (2 days) and irradiation again (10 min).

6. The influence of enzymatic reaction time on programmable assembly processes

Enzymatic conversion plays a great role in in the programmable assembly process. Herein, we examined the effect of BChE incubation time on the chromogenic performance (Figure S20). No pronounced absorbance at 638 nm was observed until incubating 95% **DC** with BChE for 12 h. From 12 h to 24 h, the absorbance representing the blue-form **PDA** increases dramatically and reaches the maximum at 24 h. After 24 h, the absorbance decreases to some extent and gets the balance at 48 h. This result indicates that incubation of 2 days is enough to hydrolyze entirely **DC** to **DA**, and then achieve the programmable self-assembly of fluorescent polymerized vesicles. The highest absorbance appeared at 24 h probably owes to the formation of co-assembly of **DA** and **DC**. To verify the assumption, we therefore screened the chromogenic performances with different mixing ratios of **DC** and **DA** (Figure S21). The chromogenic performance of **DA** firstly increases gradually along with decreasing the **DC** ratio and gets the peak at 3:7 (**DC**:**DA**), and then, the chromogenic performance decreases gradually. The result indicates that the co-assembly based on **DA** and **DC** at an appropriate ratio gave a better chromogenic performance, which is in good accordance with the result from the incubation time of BChE.

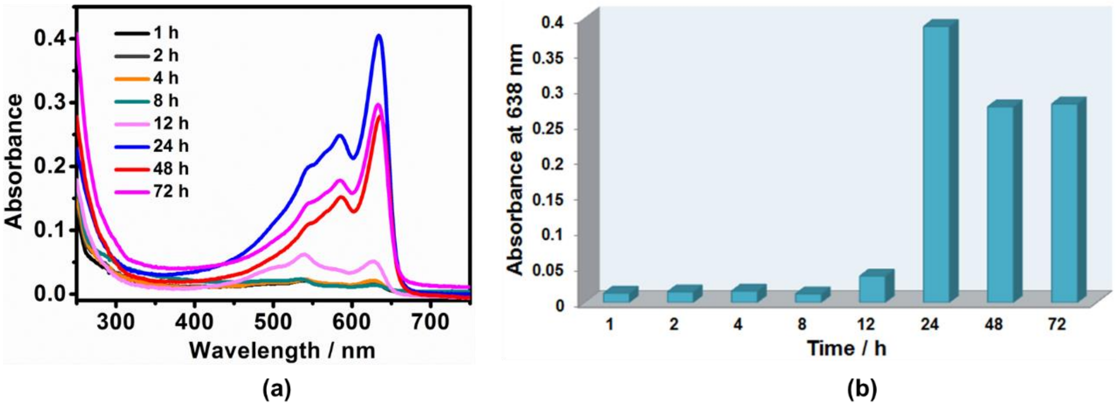

Figure S20. (a) UV-Vis spectra of 95% **DC** after incubation with BChE at 37 °C for different times in HEPES buffer (0.2 mM in diacetylene unit). Before measurements, the solution was irradiated for 5 min. (b) Dependence of the absorbance at 638 nm on the incubation time.

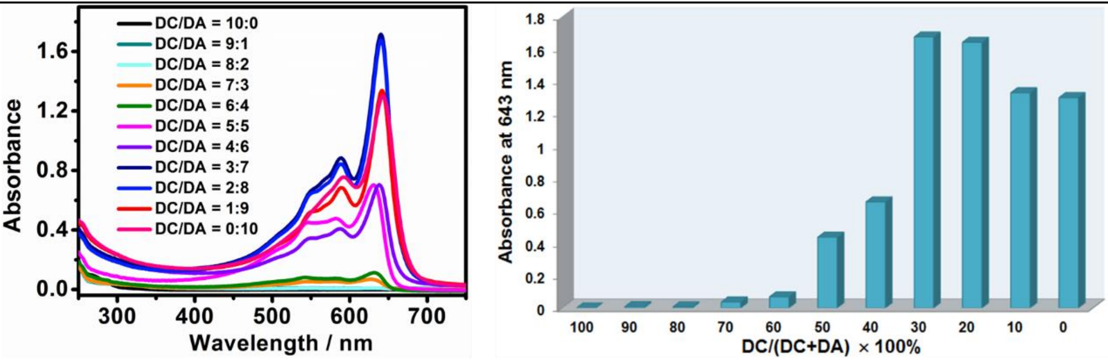

(a) (b)

Figure S21. (a) UV-Vis spectra of polydiacetylene assemblies with different mixing ratios of **DC** and **DA** in HEPES buffer (0.2 mM in diacetylene unit). Before measurements, the solution was irradiated for 5 min. (b) Dependence of the absorbance at 643 nm on the mixing molar ratio (**DC**:**DA**, from 10:0 to 0:10).

7. Programmable construction of fluorescent polymerized vesicles from DC in living cells

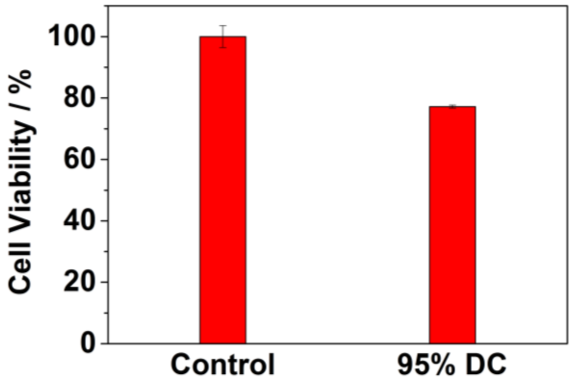

Figure S22. The HepG2 cell viability in the presence of the **DC** micelles with different **PEG**-doping ratios (0.05 mM in diacetylene unit).

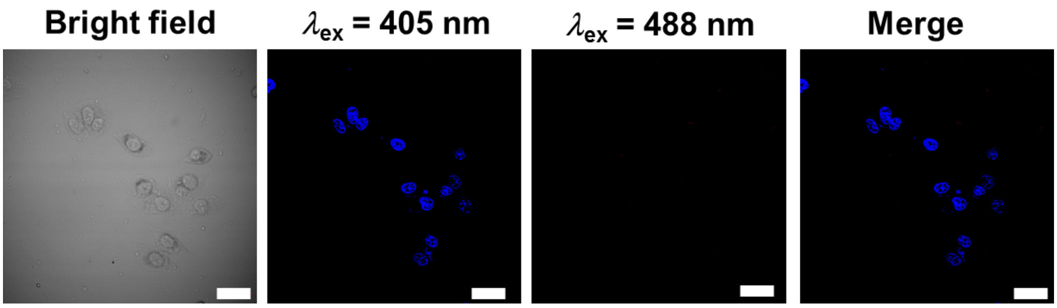

Figure S23. CLSM images of HepG2 cells stained with DAPI for 10 min at room temperature.

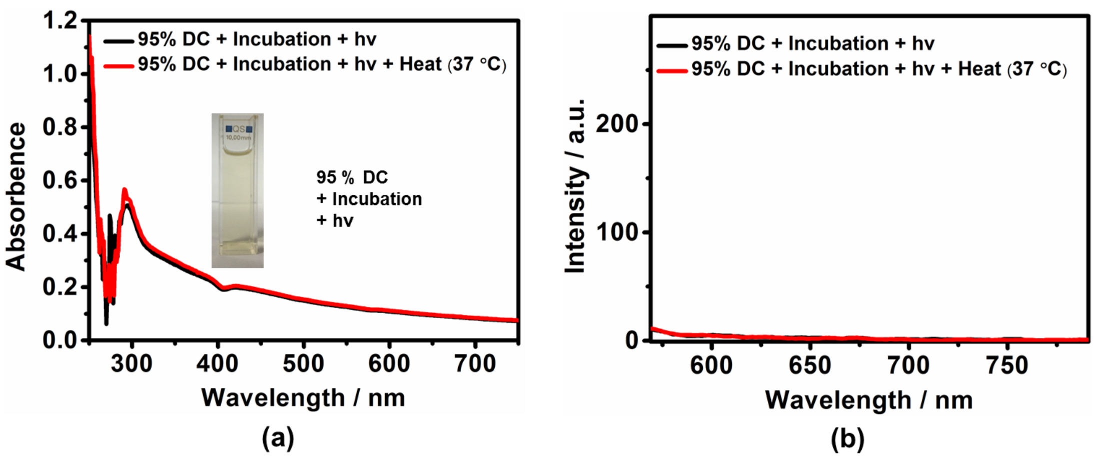

Figure S24. UV-Vis spectra (a) and fluorescence spectra (b) of 95% **DC** (0.05 mM in diacetylene unit) in DMEM supplemented with 10% FBS. Before measurements, the solution was incubated at 37 °C for 12 h, and followed by UV irradiation (254 nm) for 5 min. Inset: the photo of 95% **DC** solution after incubation and UV irradiation.

8. References

[1] H. Choi, J. S. Choi, *Bull. Korean Chem. Soc.* **2013**, *34*, 3083.

[2] X. Sun, T. Chen, S. Huang, L. Li, H. Peng, *Chem. Soc. Rev.* **2010**, *39*, 4244.

[3] M. P. Cashion, T. E. Long, *Acc. Chem. Res.* **2009**, *42*, 1016.
